# Factorial Design−Driven Optimization of Zein−Chitosan Nanoparticles for Oral Delivery of Silibinin

**DOI:** 10.1021/acsomega.5c13167

**Published:** 2026-03-06

**Authors:** Rafaelle de Sertorio dos Santos, Ariane Krause Padilha Lorenzett, Gabriela Casa Grande de Matos, Patrícia de Souza Bonfim-Mendonça, Vanderlei Aparecido de Lima, Rubiana Mara Mainardes

**Affiliations:** † Laboratory of Nanostructured Formulations, 307046 Midwest State University − UNICENTRO, Alameda Élio Antonio Dalla Vecchia St. 838, Guarapuava, Paraná 85040-167, Brazil; ‡ Pharmacy Department, Maringa State University - UEM, Avenida Colombo, St.5790, Jardim Universitário, Maringá, Paraná 87020-900, Brazil; § Chemistry Department, 74354 Federal Technological University of Paraná - UTFPR, Via do Conhecimento, s/n - KM 01 - Fraron, Pato Branco, Paraná 85503-390, Brazil

## Abstract

Silibinin (SLB) is a poorly water-soluble flavonolignan with relevant therapeutic potential but limited oral bioavailability. In this study, SLB-loaded zein−chitosan nanoparticles (SLB-ZNP) were developed by nanoprecipitation and optimized using a full 2^4^ factorial design to investigate the effects of zein concentration, chitosan concentration, incubation time, and organic-to-water ratio on particle size, polydispersity index (PDI), zeta potential, and encapsulation efficiency. The factorial approach enabled systematic evaluation of the most influential variables and their interactions, with zein and chitosan concentrations exerting major effects on particle size and surface charge, while the organic-to-water ratio significantly affected particle size distribution. The optimized formulation produced nanoparticles with a mean diameter of approximately 145 nm, low PDI (∼0.19), high positive zeta potential (∼+40 mV), and high encapsulation efficiency (∼90%). Transmission electron microscopy revealed spherical and homogeneous nanoparticles, with enhanced structural organization upon SLB incorporation. In vitro cytotoxicity assays in HeLa and SiHa cervical cancer cell lines showed that nanoencapsulation modulates carrier-associated cytotoxicity in a cell line- and concentration-dependent manner. Overall, this study demonstrates the utility of factorial design as a formulation-centered strategy for engineering zein−chitosan nanoparticles with well-defined physicochemical and in vitro properties.

## Graphical abstract

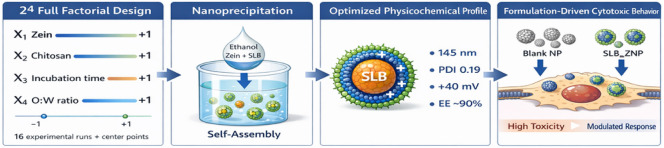

## 1 Introduction

Silibinin (SLB), also referred to as silybin or silibin, is a major bioactive constituent of the flavonolignan complex extracted from the seeds of *Silybum marianum* (milk thistle). Structurally classified as a phenolic compound, SLB consists of a mixture of two diastereomers (silybin A and silybin B) and has been extensively studied for its broad-spectrum pharmacological profile. Preclinical studies have substantiated its anti-inflammatory, hepatoprotective, antioxidant, antifibrotic, cardioprotective, and antidiabetic effects, in addition to its capacity to modulate lipid metabolism by lowering serum cholesterol levels.^1,2^ Notably, SLB has attracted significant attention in oncology due to its chemopreventive and chemotherapeutic properties. It modulates diverse signaling cascades associated with carcinogenesis, including inhibition of cell proliferation, induction of cell cycle arrest, and activation of caspase-mediated apoptotic pathways in several cancer cell lines.^3^ These pleiotropic mechanisms of action have positioned SLB as a promising candidate for adjuvant therapy in cancers of the prostate, breast, liver, lung, and colon.^4^

Despite these therapeutic prospects, the clinical translation of SLB has been hindered by two major biopharmaceutical limitations: poor aqueous solubility and inherently low oral bioavailability, which has been reported to range from 23 to 47% following conventional administration.^5^ These challenges are predominantly attributed to its hydrophobic molecular structure and extensive first-pass metabolism in the gastrointestinal tract and liver.^6^ Consequently, innovative delivery systems are required to enhance the solubility, stability, and systemic availability of SLB.

Protein-based nanoparticles have been widely investigated as drug delivery systems due to their biocompatibility, biodegradability, and structural versatility. Commonly explored proteins include albumin, gelatin, casein, whey proteins, and silk fibroin, each offering distinct advantages depending on the intended route of administration and drug properties.^7,8^ Albumin-based nanoparticles, for instance, exhibit excellent biocompatibility and binding capacity for a broad range of drugs, but often require chemical cross-linking or complex processing steps.^9^ Gelatin and casein nanoparticles are readily processable and suitable for hydrophilic compounds, yet may present limited stability under gastrointestinal conditions.^10^ In contrast, zein is a hydrophobic, prolamin-rich plant protein that spontaneously self-assembles into nanoparticles via antisolvent precipitation, forming a compact core particularly suited for the encapsulation of poorly water-soluble compounds such as SLB.^11,12^ Its α-helical and β-sheet secondary structures confer structural rigidity and resistance to proteolytic degradation, contributing to nanoparticle stability under physiological conditions.^13,14^ Moreover, its resistance to enzymatic degradation, mucoadhesive behavior, and compatibility with simple, scalable fabrication methods make zein especially attractive for oral drug delivery applications.^15,16^ These attributes, combined with the possibility of surface modification using surfactants, polysaccharides such as chitosan, motivated the selection of zein as the protein matrix in the present formulation-driven study.^17,18^

Despite the literature on SLB nanoencapsulation and its cytotoxic mechanisms at the molecular and genetic levels, most studies have focused primarily on elucidating drug-driven biological pathways, such as apoptosis induction, cell cycle arrest, and gene expression modulation, using preoptimized or empirically formulated nanocarriers.^19–22^ In contrast, comparatively little attention has been given to the systematic engineering of the carrier itself and to how formulation and process parameters govern carrier-associated biological responses. Similarly, although zein-based and zein−polysaccharide nanoparticles have been reported for oral and anticancer delivery,^23,24^ these systems are commonly developed using one-factor-at-a-time approaches, with limited evaluation of factor interactions and formulation robustness. In this context, the present study provides a distinct contribution by applying a full 2^4^ factorial design to the optimization of zein−chitosan nanoparticles encapsulating SLB, enabling quantitative assessment of the main effects and interactions of formulation variables on critical quality attributes. Rather than dissecting intracellular signaling pathways, this work emphasizes the relationship between formulation design, physicochemical properties, and carrier-driven cytotoxic behavior, offering a formulation-centered framework that complements existing mechanistic studies on SLB-loaded nanocarriers.

## 2 Experimental Section

### 2.1 Chemicals and Materials

Zein, sodium caseinate, sodium alginate, apple pectin, low molecular weight chitosan (degree of deacetylation >75%) and silibinin (SLB) were purchased from Sigma-Aldrich (St. Louis, MO, USA). Absolute ethanol and glacial acetic acid were obtained from Merck (Darmstadt, Germany). Ultrapure deionized water was produced using a Milli-Q water purification system (Millipore, Bedford, MA, USA). All reagents were of analytical grade and used without further purification.

### 2.2 Preliminary Screening of Stabilizers for Zein Nanoparticles Containing Silibinin

Before the factorial design was applied, a preliminary screening study was performed to evaluate the impact of different stabilizing agents on the physicochemical characteristics of zein-based nanoparticles loaded with SLB (SLB-ZNP). This preformulation stage aimed to identify the most appropriate stabilizer to be used in the subsequent optimization step.

Nanoparticles were prepared using the nanoprecipitation method with modifications adapted from the protocol described by Galindo-Rodriguez et al.^25^ Initially, 30 mg of zein was dissolved in 3 mL of 85% (v/v) aqueous ethanol under magnetic stirring for 1 h at room temperature. In parallel, SLB was solubilized in absolute ethanol at a concentration of 3.5 mg/mL. The aqueous phase consisted of the stabilizer dissolved in either water or 1.5% acetic acid, depending on its solubility.

The following stabilizers were evaluated: sodium caseinate at 40 mg, apple pectin at 3.5 mg, sodium alginate at 20 mg, a combination of pectin and alginate at 24 mg and 3.6 mg respectively, and chitosan at 3 mg dissolved in 1.5% (v/v) acetic acid. The ethanolic SLB solution was then added to the zein hydroalcoholic solution, and the resulting organic phase was gently stirred at 150 rpm and 25 °C for 30 min in an orbital incubator. This mixture was subsequently poured into the aqueous stabilizer solution under continuous agitation, promoting nanoparticle formation through spontaneous desolvation.

After nanoprecipitation, ethanol was removed using a rotary evaporator operating at 300 rpm and 40 °C for 5 min. The resulting nanosuspension was centrifuged at 2,520 × *g* and 25 °C for 30 min to separate the nonencapsulated drug and excess stabilizer. The supernatant and pellet were both collected for further analysis. Blank nanoparticles were prepared under identical conditions, excluding the drug. This preliminary screening enabled the selection of the optimal stabilizer based on mean particle diameter, polydispersity index (PDI), zeta potential and encapsulation efficiency (EE).

The physicochemical parameters of the nanoparticles obtained with each stabilizer were statistically evaluated through one-way analysis of variance (ANOVA), followed by Tukey’s post hoc test to determine significant pairwise differences between formulations. Statistical analyses were conducted using Minitab version 18, with a significance threshold of *p* < 0.05. The comparison focused specifically on the formulations prepared with different stabilizers, evaluating mean particle size, PDI, zeta potential, and EE. Based on these outcomes, the stabilizer that achieved the most balanced and favorable profile across all parameters, characterized by reduced particle size, lower PDI, higher surface charge stability, and improved EE, was selected for subsequent optimization experiments.

### 2.3 Optimization of Zein Nanoparticle Formulations through Factorial Experimental Design

After the preliminary screening and selection of chitosan as the stabilizing agent, the nanoprecipitation method described in Section 2.2 was applied using a structured experimental design to optimize the formulation. A full two-level 2^4^ factorial design was employed to systematically investigate the effects of four independent variables: zein concentration (X_1_), chitosan concentration (X_2_), incubation time in the orbital shaker (X_3_), and organic-to-water ratio (O:W) (X_4_). All possible combinations of factor levels were experimentally evaluated, characterizing a nonfractional factorial design. The ranges selected for independent variables were established based on preliminary screening experiments. These initial studies were conducted to delimit conditions that allowed reproducible nanoparticle formation, adequate colloidal stability, and reliable experimental handling, while avoiding regimes associated with precipitation or excessive aggregation.

The design comprised 16 factorial points supplemented with six center points, resulting in a total of 22 experimental runs, which were conducted in randomized order. The center points consisted of independent replicated preparations and were included to estimate pure experimental error and assess process robustness. Each experimental run corresponded to an independent nanoparticle batch, and all response variables, mean particle diameter (R_1_), PDI (R_2_), zeta potential (R_3_), and EE (R_4_) were determined from these independent preparations. Physicochemical measurements were performed in triplicate for each batch, and results are reported as mean ± standard deviation.

The experimental design and statistical analyses were carried out using Minitab version 18. ANOVA and multiple linear regression were applied to identify significant main effects and interaction terms, as well as to evaluate model adequacy. The investigated factor levels and response variables are summarized in Table 1. Independent variables were evaluated at two levels and coded as −1 (low) and +1 (high), with center points coded as 0. Regression analyses and model construction were performed using the coded variables, whereas the corresponding actual factor values are reported in their original units.

**Table 1:** Independent Variables and Experimental Levels for the Factorial Design in the Development of Zein−Chitosan Nanoparticles

		Levels		
Factor	Independent variables	−1	0	+1
X_1_	Zein concentration (% m/V)	0.02	0.03	0.04
X_2_	Chitosan concentration (% m/V)	0.003	0.0075	0.012
X_3_	Incubation time (min)	30	75	120
X_4_	O:W ratio (v/v)	1:1	1:2	1:3
Dependent variables				
R_1_	Average diameter			
R_2_	Polydispersity index			
R_3_	Zeta potential			
R_4_	Encapsulation efficiency			

The optimization criteria were defined based on established requirements for oral nanoparticle drug delivery systems. A target particle diameter below 300 nm was selected to favor intestinal transport and cellular uptake, while minimizing aggregation and premature clearance.^26^ A PDI lower than 0.2 was adopted as an indicator of narrow size distribution and colloidal homogeneity.^27^ Zeta potential values with absolute magnitude ≥30 mV were considered desirable to ensure electrostatic stabilization of the nanosuspension.^28^ EE was defined as high when EE ≥ 80%, a threshold commonly associated with efficient drug loading and reduced drug loss during nanoparticle preparation.^29^ These criteria are consistent with widely accepted standards reported for orally administered nanocarriers.

### 2.4 Physicochemical Characterization

#### 2.4.1 Mean Particle Diameter and Polydispersity Index (PDI)

The average hydrodynamic diameter and PDI of SLB-ZNP were measured by dynamic light scattering (DLS) using a Zetasizer Nano-ZS ZEN3600 instrument (Malvern Instruments Ltd., Worcestershire, UK), which operates based on DLS coupled with particle electrophoresis. For analysis, an aliquot of the nanoparticle suspension was diluted in ultrapure water at a volumetric ratio of 1:200 (v/v) and transferred to a quartz cuvette. Measurements were performed at 25 °C with a fixed scattering angle of 90°, using a 659 nm laser source. All determinations were conducted in triplicate, and results are expressed as mean ± standard deviation.

#### 2.4.2 Zeta Potential

Zeta potential values of SLB-ZNP were determined by laser Doppler electrophoresis using the Zetasizer Nano-ZS ZEN3600. Samples were diluted at a 1:200 (v/v) ratio in a 1 mM KCl solution to maintain consistent ionic strength. Analyses were conducted at 25 °C under an applied voltage of ±150 mV in an electrophoretic cell. Measurements were performed in triplicate and are presented as mean ± standard deviation.

#### 2.4.3 Transmission Electron Microscopy (TEM) Analysis

Morphological characterization was performed by TEM using a JEOL JEM-1400 Plus microscope operating at 100 kV. Samples were diluted 1:100 in ultrapure water (18.2 MΩ·cm), and 10 μL of the suspension was deposited onto commercial 300-mesh nickel grids coated with Formvar/carbon film (Ted Pella Inc., 01753N-F). Grids were dried at room temperature prior to analysis. Images were acquired from representative sample regions. Particle size was measured directly from micrographs using the TEM Center for JEM-1400 Plus software. For comparison and validation, additional measurements were performed in ImageJ. A minimum of 100 particles per sample was analyzed to ensure statistical reliability.

### 2.5 Encapsulation Efficiency (EE)

EE was determined indirectly by quantifying the amount of nonencapsulated SLB in the supernatant following centrifugation of the SLB-ZNP suspension. After centrifugation, an aliquot of the supernatant was collected, and the amount of SLB present in this fraction (SLB_supernatant_) was quantified by UV−Vis spectrophotometry at 288 nm using a previously validated calibration curve. The encapsulated amount of SLB was calculated by difference, considering SLB_added_ as the initial amount of SLB introduced into the formulation during nanoparticle preparation. EE was calculated according to eq 1:

1 $$\mathrm{EE\%}=\left(\frac{{\mathrm{SLB}}_{\mathrm{added}}-{\mathrm{SLB}}_{\mathrm{supernatant}}}{{\mathrm{SLB}}_{\mathrm{added}}}\right)\;\times \;100$$

where SLB_added_ and SLB_supernatant_ are expressed in the same units. Results are reported as mean ± standard deviation (n = 3).

### 2.6 Cytotoxicity Over Tumor Cells

HeLa (human cervical adenocarcinoma) and SiHa (human cervical squamous carcinoma) cells (2 × 10^5^ cells/well) were cultured in Dulbecco’s Modified Eagle Medium (DMEM) supplemented with 10% fetal bovine serum and 1% penicillin−streptomycin−amphotericin B. Cultures were maintained at 37 °C in a humidified 5% CO_2_ atmosphere for 24 h. At approximately 90% confluence, cells were detached with trypsin, seeded into 24-well plates, and allowed to adhere overnight.

Cells were then exposed to SLB-ZNP, blank ZNP, or free SLB at final concentrations ranging from 790 to 3161 μM for 24 and 48 h. Untreated cells maintained in complete medium served as controls. All tested concentrations are expressed as SLB-equivalent (μM) for both free SLB and SLB-ZNP, allowing direct comparison between formulations. For ZNP, concentrations correspond to the equivalent nanoparticle mass used in SLB-ZNP formulations. Cell viability was determined after 24 and 48 h using the MTS assay. Following treatment, cells were washed with phosphate-buffered saline, and phenol-red−free DMEM containing 100 μL of MTS reagent (3-(4,5-dimethylthiazol-2-yl)-5-(3-carboxymethoxyphenyl)-2-(4-sulfophenyl)-2H tetrazolium) was added. Plates were incubated for 3 h at 37 °C, and absorbance was measured at 490 nm using a SpectraMax Plus 384 microplate reader. Cell viability was calculated according to eq 2. Results are expressed as mean ± standard deviation (n = 2). Statistical significance was assessed using one-way ANOVA followed by Tukey’s post hoc test (GraphPad Prism 8.0).

2 $$\mathrm{Cell viability}\;(\%)=\left(\frac{\mathrm{Absorbance of treated cells}}{\mathrm{Absorbance of control}}\right)\;\times \;100$$

## 3 Results and Discussion

### 3.1 Screening of Stabilizers for SLB-ZNP Formulation

In the preformulation stage, five different stabilizers were evaluated to identify the most suitable candidate for the preparation of SLB-ZNP via nanoprecipitation. The stabilizers tested included sodium caseinate, sodium alginate, apple pectin, a combination of pectin and alginate, and low molecular weight chitosan. Each formulation was characterized in terms of mean particle size, PDI, zeta potential, and EE, as summarized in Table 2.

**Table 2:** Physicochemical Characteristics of SLB-Loaded Zein-Based Nanoparticles Prepared with Different Stabilizers[Table-fn tbl2fn1]

Stabilizer	Mean particle size (nm)	PDI	Zeta potential (mV)	EE (%)
Caseinate	165.8^a^	0.119^a^	−25^c^	68.4^b^
Alginate	218.9^b^	0.156^a^	−36^a^	92.9^a^
Pectin	300.5^b^	0.125^a^	−33^a^	93.5^a^
Pectin + Alginate	320.9^b^	0.168^a^	−35^a^	94.1^a^
Chitosan	171.7^a^	0.148^a^	+44^b^	95.4^a^

i Different letters in the same column indicate statistically significant differences (*p* <0.05) according to Tukey’s test.

All formulations yielded nanoparticles with sizes below 350 nm and PDI values under 0.2, indicating monodisperse systems and acceptable homogeneity. However, notable differences were observed among the stabilizers in terms of particle size and surface charge. Sodium caseinate and chitosan led to significantly smaller particle sizes, with average diameters of 165.8 and 171.7 nm, respectively, which were statistically lower than those obtained with alginate (218.9 nm), pectin (300.5 nm), and the pectin−alginate combination (320.9 nm) (*p* < 0.05).

Regarding zeta potential, formulations stabilized with alginate, pectin, or their combination exhibited negative surface charges ranging from −33 mV to −36 mV, consistent with the anionic nature of these polysaccharides. Conversely, chitosan-stabilized nanoparticles displayed a markedly positive zeta potential of +44 mV, attributed to the protonated amino groups of chitosan in acidic medium.^30^ The formulation with caseinate showed an intermediate value for zeta potential (−25 mV), suggesting a moderate surface charge.

EE was significantly influenced by the type of stabilizer. Chitosan provided the highest EE (95.4%), followed by the pectin−alginate blend (94.1%), pectin alone (93.5%), and alginate (92.9%), all of which were statistically similar (*p* > 0.05). In contrast, caseinate resulted in a significantly lower EE (68.4%), suggesting suboptimal interaction with the hydrophobic SLB molecule during nanoparticle formation.

Importantly, only the chitosan-based formulation achieved an optimal balance between small particle size, narrow size distribution, high EE, and a strongly positive zeta potential. The latter is particularly advantageous for enhancing mucoadhesion and facilitating interaction with negatively charged mucosal membranes, which is desirable for oral delivery applications.^31,32^

Overall, these results demonstrate that chitosan is the most promising stabilizer for the formulation of SLB-ZNP, owing to its distinctive cationic nature and excellent biocompatibility. These properties are particularly well-suited to improve nanoparticle stability and foster favorable biological interactions. These attributes strongly support its selection for subsequent optimization through factorial design in the next stage of this study.

### 3.2 2^4^ Factorial Design Optimization of SLB-ZNP via Nanoprecipitation

The objective of the 22 experiments was to evaluate the effect of process-independent variables (factors) on four response variables: mean diameter (R_1_), PDI (R_2_), zeta potential (R_3_), and SLB EE (R_4_). Six experiments were performed as center points (F3) with repeated concentrations allowing estimation of pure experimental error. Complete data of the experimental design are provided in Table 3. After data collection, each response was individually analyzed to determine how changes in factor levels influenced the outcomes. Mathematical modeling was conducted in Minitab using multiple regression and, ANOVA. These procedures ensured the reliability of the model and allowed assessment of regression significance and goodness of fit.

**Table 3:** Response Variables Obtained for the Optimization of SLB-ZNP by the Nanoprecipitation Method^a^

Formulation	R_1_	R_2_	R_3_	R_4_
F1	178 ± 22	0.40 ± 0.13	+44.0 ± 1.7	82.0 ± 0.1
F2	274 ± 175	0.40 ± 0.03	+30.0 ± 11.4	60.0 ± 0.1
F3	181 ± 18	0.30 ± 0.05	+36.0 ± 2.6	90.00 ± 0.01
F3	174 ± 22	0.20 ± 0.04	+39.0 ± 0.7	85.00 ± 0.01
F3	206 ± 18	0.10 ± 0.04	+10.0 ± 1.3	95.00 ± 0.01
F3	200 ± 18	0.10 ± 0.04	+35.0 ± 8.1	97.0 ± 0.0
F3	163 ± 6	0.30 ± 0.06	+40.0 ± 2.0	87.00 ± 0.05
F3	228 ± 35	0.50 ± 0.06	+38.0 ± 1.6	0.0 ± 0.0
F4	242 ± 80	0.60 ± 0.08	+35.0 ± 5.3	74.00 ± 0.08
F5	331 ± 14	0.10 ± 0.08	+39.0 ± 0.6	90.00 ± 0.01
F6	1655 ± 542	0.40 ± 0.04	+48.0 ± 0.2	0.0 ± 0.0
F7	387 ± 33	0.10 ± 0.00	+41.0 ± 1.0	96.00 ± 0.01
F8	223 ± 35	0.60 ± 0.13	+27.0 ± 1.6	78.00 ± 0.05
F9	171 ± 52	0.40 ± 0.15	+13.0 ± 8.8	78.00 ± 0.04
F10	168 ± 38	0.30 ± 0.04	+30.0 ± 1.4	66.00 ± 0.09
F11	209 ± 9	0.30 ± 0.04	+39.0 ± 1.7	78.00 ± 0.11
F12	1581 ± 563	0.60 ± 0.10	+47.0 ± 2.0	0.0 ± 0.0
F13	1504 ± 148	0.50 ± 0.00	+46.0 ± 2.0	0.0 ± 0.0
F14	304 ± 39	0.10 ± 0.00	+42.0 ± 0.9	0.0 ± 0.0
F15	240 ± 2	0.09 ± 0.01	+39.0 ± 0.5	98.0 ± 0.0
F16	823 ± 140	0.50 ± 0.00	+45.0 ± 1.9	98.0 ± 0.0
F17	602 ± 141	0.90 ± 0.04	+37.0 ± 3.8	97.00 ± 0.02

a Response variables are expressed as R_1_ (mean size), R_2_ (PDI), R_3_ (zeta potential), and R_4_ (SLB encapsulation efficiency). Runs were performed in randomized order. Six independent center-point replicates (F3) are grouped for clarity.

#### 3.2.1 Influence of Formulation Variables on Particle Size

The mean particle diameter of SLB-ZNP was significantly influenced by multiple formulation parameters, as revealed by the results of the 2^4^ full factorial design. The O:W ratio and stabilizer concentration were identified as the most impactful variables, both exhibiting statistically significant effects at the 95% confidence level. Specifically, the O:W ratio (Factor 4) exerted a strong negative effect (−4.52), indicating that increasing the aqueous phase volume favored the formation of smaller nanoparticles (Figure 1a). In contrast, increasing the stabilizer concentration (Factor 2) resulted in a significant increase in particle size (+4.35), potentially due to higher viscosity and reduced desolvation efficiency during nanoprecipitation.^33^

**Figure 1:**
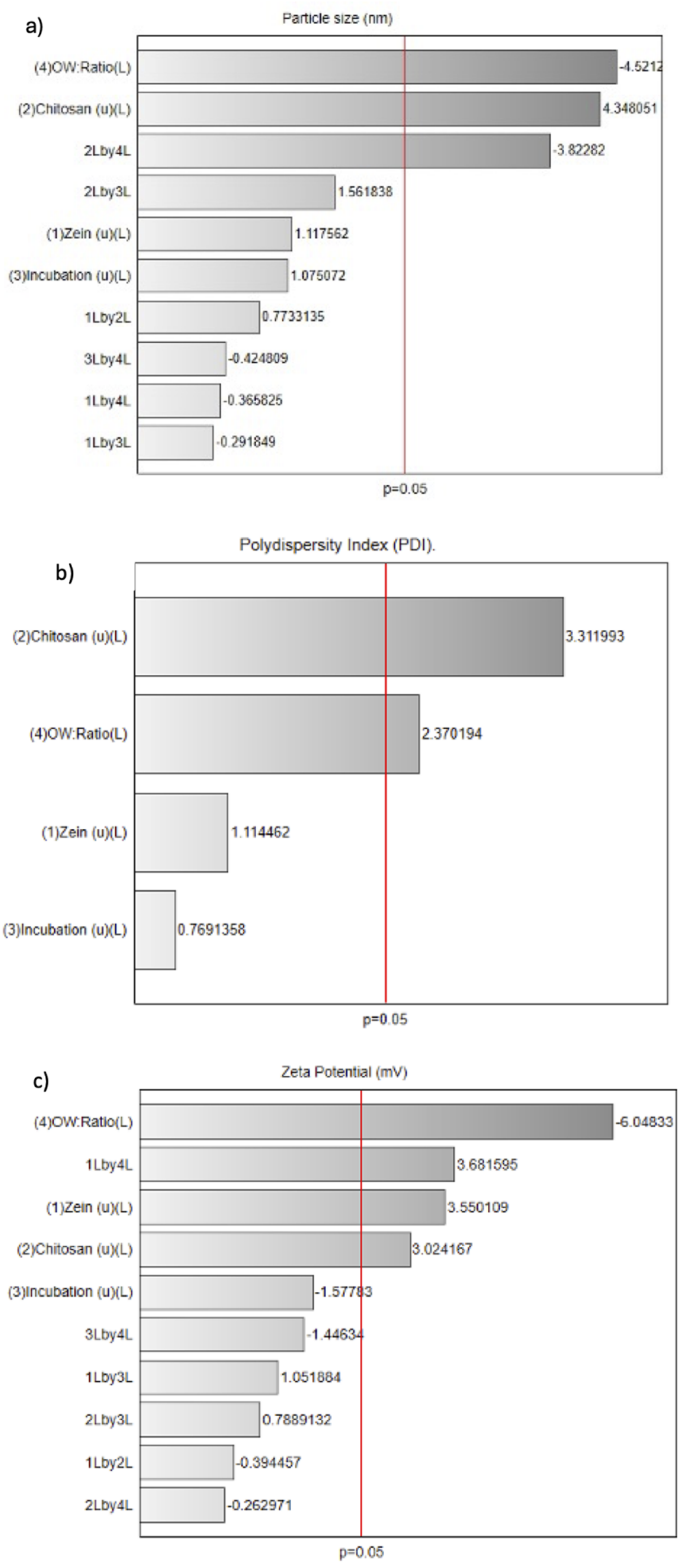
Pareto charts showing the standardized effects on (a) particle size, (b) PDI, and (c) zeta potential (*p* < 0.05).

A statistically significant negative interaction between stabilizer concentration and the O:W ratio (−3.82) was also observed, indicating that simultaneously reducing both variables produces a synergistic effect on particle size reduction. This suggests that lower stabilizer levels, when combined with a reduced O:W ratio, enhance molecular packing and favor the formation of smaller, more uniform nanoparticles. Additionally, although less pronounced, interaction effects were detected for incubation time and zein concentration, which may further influence nanoparticle assembly dynamics, albeit to a lesser extent.

To mathematically model these observations, a linear regression model including two-level interactions was derived and found to be statistically robust. The model for particle size is presented in eq 3:

3 $$\mathrm{PS}=495.352+289{.019X}_{2}-297{.414X}_{4}-251{.469X}_{2}X_{4}$$

where PS represents the predicted particle size (nm), and X_2_ and X_4_ correspond to the coded values of chitosan concentration and O:W ratio, respectively.

The model exhibited a coefficient of determination (R^2^) of 88.20%, indicating that it explains most of the variability in the response variable. ANOVA supported the model’s validity, with a calculated F-value of 5.98 exceeding the critical F-value of 3.34 (α = 0.05), confirming the statistical significance of the model. Among the variables, chitosan concentration showed a particularly strong individual effect, with a p-value of 0.0024, reinforcing its key role in determining particle size.

The adjusted coefficient of determination (R^2^_adj_) was 73.45%, indicating good explanatory power after correction for the number of predictors. The overall regression was statistically significant (overall F-test, *p* < 0.05), and the lack-of-fit test was not significant (*p* > 0.05), confirming the adequacy of the model within the studied experimental domain.

Residual analysis further validated the model’s assumptions. The normal probability plot and residual histogram (Figure S1) confirmed the normality and homoscedasticity of the residuals, with no evidence of systemic bias or variance heterogeneity.

The response surface plots (Figure 2a−c) visually reinforced the findings of the regression analysis. A clear trend of increasing particle size is observed with higher concentrations of both zein and chitosan (Figure 2a). Figure 2b demonstrates a similar pattern with increasing incubation time and zein concentration. Figure 2c highlights the dominant influence of the O:W ratio, where lower organic fractions (i.e., higher aqueous volumes) consistently resulted in smaller particles across all levels of zein.

**Figure 2:**
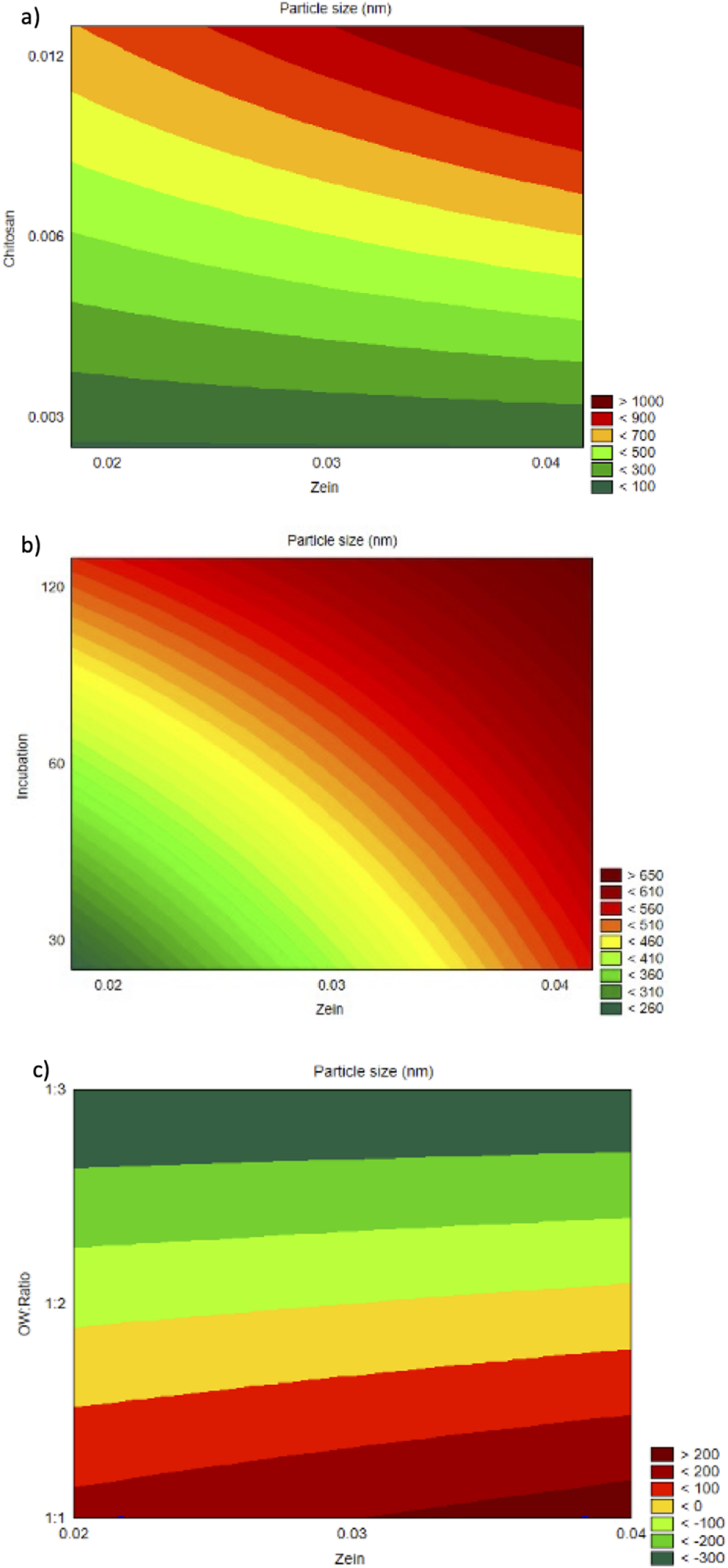
Response surface contour plots of particle size as a function of: (a) zein (%) and chitosan (%); (b) zein (%) and incubation time; (c) zein (%) and O:W ratio (v/v).

Collectively, the results suggest that the formation of smaller and more monodisperse SLB-ZNP is favored under conditions of lower zein and chitosan concentrations, reduced incubation time, and higher aqueous phase volumes. These findings are consistent with the mechanisms of nanoprecipitation, wherein rapid desolvation and diffusion gradients drive the formation of compact nanostructures.^34,35^ Excess stabilizer or high organic content can hinder this process by altering interfacial tension and particle aggregation dynamics, ultimately compromising nanoparticle uniformity and stability.^36,37^

#### 3.2.2 Influence of Formulation Variables on PDI

PDI is an important indicator of the homogeneity of nanoparticle populations, with lower values indicating more uniform size distributions.^27,38^ In this study, the PDI of SLB-ZNP formulations was influenced by formulation parameters, as assessed through the factorial design.

The Pareto chart (Figure 1b) identified chitosan concentration and the O:W ratio as the main factors affecting PDI. Chitosan exhibited the strongest positive effect (+3.31), followed by the O:W ratio (+2.37), both surpassing the statistical threshold at the 95% confidence level. In contrast, variations in zein concentration and incubation time did not show significant effects on PDI within the tested design space.

To better understand the relationship between these variables and PDI, a linear regression model with main effects was derived and is presented in eq 4:

4 $$\mathrm{PDI}=0.3731+0{.1318X}_{2}+0{.097X}_{4}$$

where PDI represents the predicted polydispersity index, and X_2_ and X_4_ correspond to the coded values of chitosan concentration and O:W ratio, respectively.

This model presented an R^2^ of 56.80%, indicating a moderate yet acceptable ability to explain the variability in PDI. Despite the lower R^2^ compared to particle size modeling, the model was statistically significant, as confirmed by ANOVA. The calculated F-value of 4.605 exceeded the critical value of 3.347 (α = 0.05), validating the model’s statistical significance.

The adjusted R^2^ of the PDI model was 44.48%, and the overall regression was statistically significant (overall F-test, *p* < 0.05), with no significant lack of fit (*p* > 0.05). However, the moderate R^2^ value indicates limited predictive capability. Therefore, this model was primarily used to identify general trends and the direction of factor effects, and PDI predictions should be interpreted with caution during optimization.

Residual analysis (Figure S2) confirmed that the assumptions of normality and homoscedasticity were met, with residuals displaying a symmetric distribution around zero and no evidence of heteroscedasticity.

The response surface plots (Figure 3a−c) further corroborated these findings and illustrate the influence of chitosan and the O:W ratio on the PDI variable. As shown in Figure 3a, PDI increased with rising chitosan concentrations, indicating greater size heterogeneity, potentially due to the formation of polymeric networks or bridging flocculation under higher polycationic densities. Figure 3b demonstrated a similar increase in PDI with higher O:W ratios, possibly due to slower diffusion and less efficient nucleation. Meanwhile, Figure 3c confirmed the limited influence of incubation time on PDI, consistent with the nonsignificant statistical contribution of this factor.

**Figure 3:**
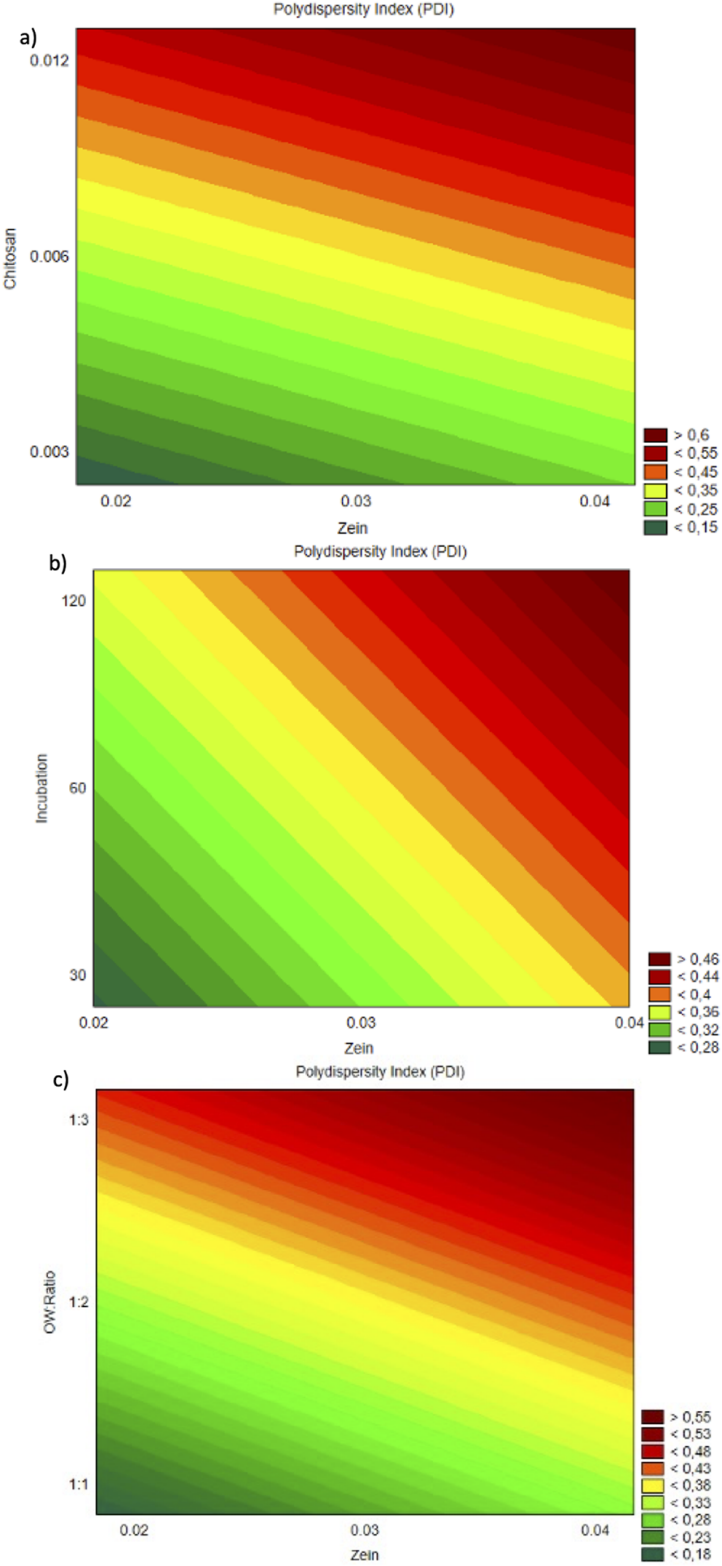
Response surface contour plots of PDI as a function of: (a) zein (%) and chitosan (%); (b) zein (%) and incubation time; (c) zein (%) and O:W ratio (v/v).

Taken together, the results suggest that maintaining lower concentrations of chitosan and a reduced O:W ratio is essential to minimize PDI and promote the formation of monodisperse SLB-ZNP. These findings align with the physicochemical expectations for nanoprecipitation processes, where rapid mixing and lower polymer concentrations favor uniform nucleation and controlled particle growth.^39,40^

#### 3.2.3 Influence of Formulation Variables on Zeta Potential

Zeta potential is a key physicochemical parameter used to evaluate the surface charge and electrostatic stability of nanoparticle suspensions. A higher absolute zeta potential generally indicates greater colloidal stability due to increased repulsive forces between particles.^41^ In the present study, the zeta potential of SLB-ZNP was found to be significantly affected by multiple formulation variables, as revealed by the factorial design analysis.

The Pareto chart (Figure 1c) identified the O:W ratio as the most influential factor on zeta potential, with a highly significant negative effect (−6.05). This was followed by positive contributions from zein concentration (+3.55) and chitosan concentration (+3.02), while incubation time did not exhibit statistical significance under the studied conditions.

To quantitatively model these effects, a linear regression model with two-level interactions was developed. The resulting predictive equation for zeta potential (ZP) is shown in eq 5:

5 $$\mathrm{ZP}=37.7368+3{.3750X}_{1}+2{.8750X}_{2}-5{.7500X}_{4}+3{.5000X}_{1}X_{4}$$

where ZP represents the predicted zeta potential (mV), and X_1_, X_2_, and X_4_ correspond to the coded values of zein concentration, chitosan concentration, and O:W ratio, respectively.

This model demonstrated a high coefficient of determination (R^2^ = 90.74%), indicating excellent predictive power and strong correlation between the experimental data and the model. ANOVA further supported its robustness, with an F-value of 7.842, well above the critical value of 3.347 at α = 0.05, confirming the statistical significance of the model.

Among the variables, the O:W ratio had the most substantial influence, indicating that increasing the organic-to-water ratio decreases the zeta potential significantly. Both zein concentration and chitosan concentration exert significant positive effects (+3.55 and +3.02, respectively), enhancing the zeta potential. The positive effect of chitosan is attributed to its cationic nature under acidic conditions, contributing positively charged amine groups to the nanoparticle surface.^42^ The zein influence may be related to its amino acid composition and the orientation of side chains during nanoparticle self-assembly.^43^

The response surface plots (Figure 4a−c) confirmed these trends. The zeta potential increased with higher concentrations of zein and chitosan, reaching values above +44 mV (Figure 4a). Similarly, Figure 4b shows that shorter incubation times combined with high zein concentrations also favored higher surface charges. Figure 4c demonstrates that lower O:W ratios resulted in markedly higher zeta potentials, confirming the dominance of this factor in modulating the electrokinetic behavior of the system.

**Figure 4:**
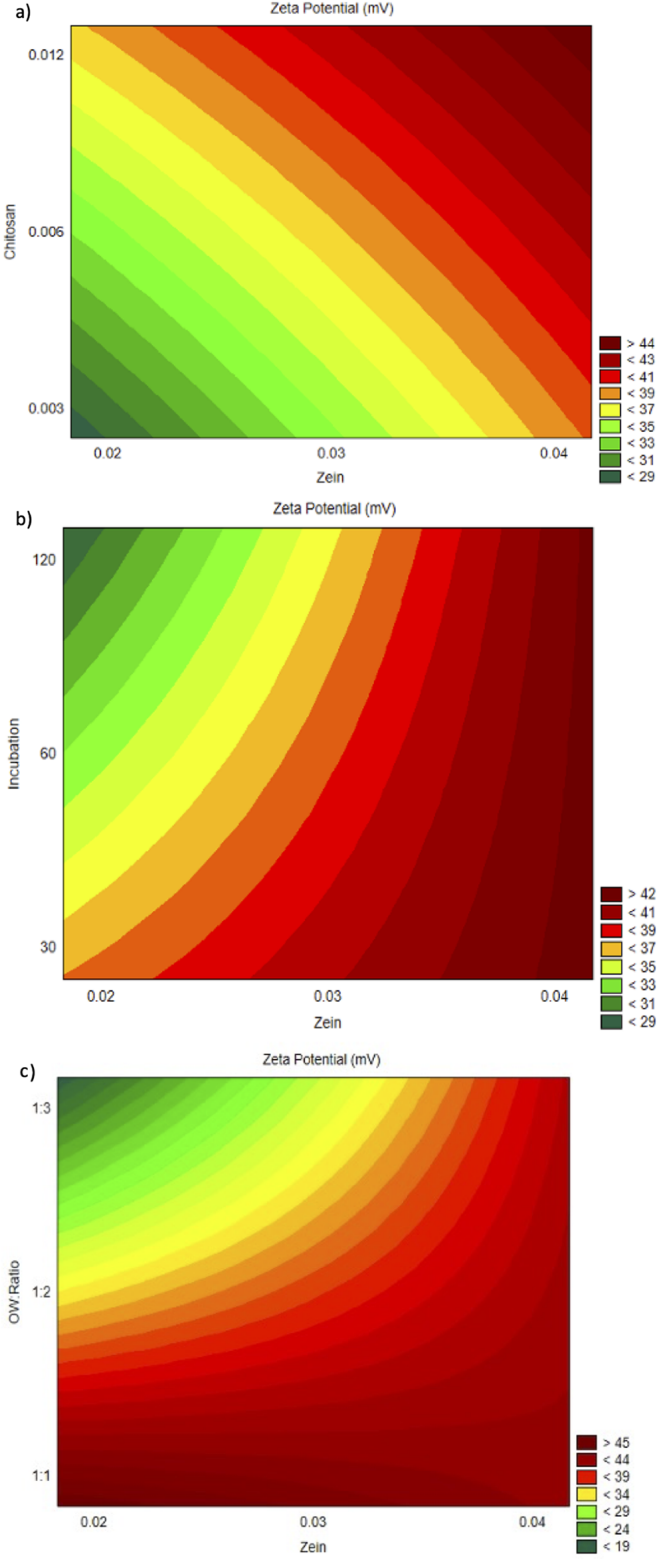
Response surface contour plots of zeta potential as a function of: (a) zein (%) and chitosan (%); (b) zein (%) and incubation time; (c) zein (%) and O:W ratio (v/v).

The residual analysis (Figure S3) indicated an adequate fit of the model, with residuals normally distributed and randomly scattered, supporting the validity of the regression model.

Overall, these results confirm that modulating the O:W ratio is the most effective strategy to control the zeta potential of SLB-ZNP, followed by adjustment of zein and chitosan concentrations. Maintaining higher surface charge values is crucial to ensure colloidal stability and prevent particle aggregation during storage and administration of the nanoparticles.^28^

#### 3.2.4 Influence of Formulation Variables on Encapsulation Efficiency

The EE of SLB-ZNP formulations was evaluated to determine the extent to which SLB was retained within the nanoparticle matrix following nanoprecipitation. However, the statistical analysis revealed that the tested formulation variables did not significantly influence EE within the studied range. Given the nonsignificant regression and low explanatory power of the model, EE was not predicted using regression equations. Instead, EE was treated as an experimental response, and only experimentally measured mean values were considered in subsequent optimization steps.

The model failed to account for the variability in EE across the experimental conditions, suggesting that the factors investigated, including zein concentration, chitosan concentration, O:W ratio, and incubation time, did not exert a statistically significant effect on the encapsulation of SLB under the conditions tested.

The application of a linear model followed by ANOVA resulted in a low coefficient of determination (R^2^ = 20.61%), indicating limited explanatory power of the model for EE. This outcome suggests that, within the investigated design space, the selected formulation and process variables, namely zein concentration, chitosan concentration, O:W ratio, and incubation time, were not the primary determinants of SLB encapsulation. Instead, EE is likely governed by factors not explicitly captured by the factorial design, such as molecular-level drug−polymer affinity, intermolecular interactions, and kinetic phenomena during nanoprecipitation. Accordingly, EE was interpreted as an experimentally determined response rather than a parameter suitable for predictive modeling in the present study.

Due to the nonsignificance of the model and the absence of meaningful trends, no response surface plots or Pareto charts were included for EE, in accordance with good statistical practice. This result suggests that SLB entrapment in the zein matrix is relatively robust to formulation changes within the tested design space, possibly due to its strong affinity for the hydrophobic domains of the protein, which may promote spontaneous incorporation independent of minor variations in process parameters.^36^

These findings reinforce the formulation-driven scope of the present study and indicate that modulation of EE would likely require exploration of additional variables or expanded experimental domains

### 3.3 Multivariate Analysis of SLB-ZNP Formulations

To complement the factorial analysis and better understand the interrelationships between the response variables and formulation groups, multivariate statistical techniques were applied, including Principal Component Analysis (PCA) and Hierarchical Cluster Analysis (HCA).

PCA was conducted using the main physicochemical parameters (mean particle size, PDI, zeta potential, and EE), allowing dimensionality reduction and visualization of latent patterns among the experimental formulations. The resulting biplot (Figure 5) revealed that the first two principal components explained 80.9% of the total variance in the data set (PC1 = 54.2% and PC2 = 26.7%).

**Figure 5:**
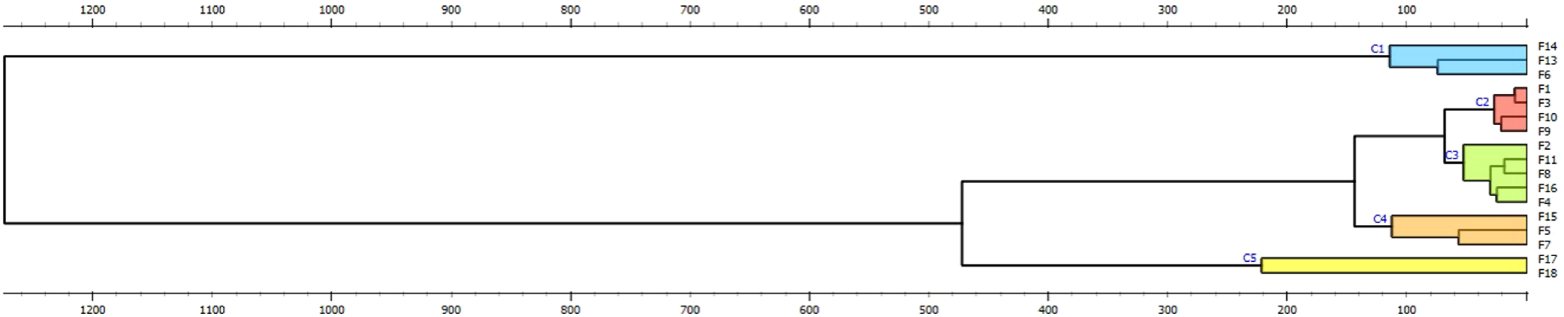
Hierarchical clustering dendrogram of nanoparticles formulations based on the dependent variables.

The loading vectors indicated that zeta potential and particle size were positively correlated and aligned along PC1, whereas EE projected in the opposite direction. The PDI vector showed a distinct orientation, suggesting partial independence from the other variables. These results indicate that formulations with higher surface charge and larger particle size tend to exhibit lower EE, whereas samples with low PDI cluster near the origin, reflecting greater homogeneity.

The hierarchical clustering analysis, performed using Euclidean distance and Ward’s linkage method, revealed five well-defined groups of formulations (Figure 6). The clusters were distributed as follows: C1 (F6, F13, F14), C2 (F1, F3), C3 (F2, F8, F9, F11), C4 (F4, F15, F16), and C5 (F5, F7, F18, F17). This distribution highlights distinct similarity patterns, with C1 and C2 positioned in proximity, reflecting a high degree of internal homogeneity, whereas C5 was markedly divergent, forming a separate branch apart from the other groups.

**Figure 6:**
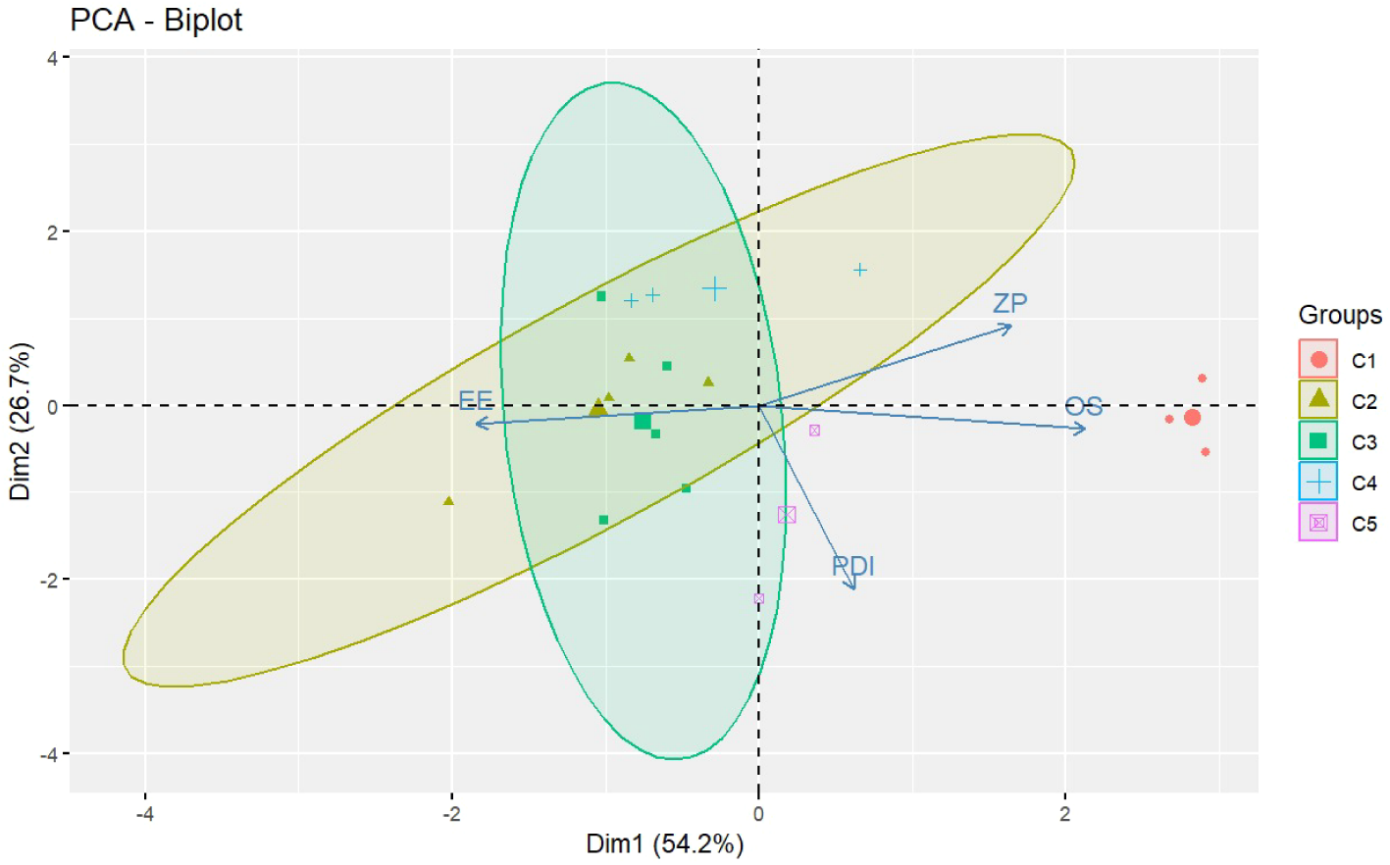
PCA biplot showing the projection of the dependent variables (loadings) and formulations (scores), illustrating their interrelationships.

These results indicate that formulations with comparable physicochemical attributes, particularly those optimized for higher zeta potential and smaller particle size (e.g., C3 and C4), tended to cluster together. The consistency observed between the dendrogram and PCA outcomes reinforces the discriminatory power of the factorial design and confirms that the experimental conditions effectively guided the formation of distinct formulation groups.

Together, PCA and HCA provided a deeper understanding of the relationships between formulation variables and nanoparticle characteristics, confirming that multivariate analysis can effectively distinguish between experimental conditions and support the formulation design in nanoparticle systems.^44^

### 3.4 Response Optimization

The optimization of the SLB-ZNP was performed using a multiresponse desirability function (Derringer−Suich approach), aiming to simultaneously achieve predefined quality targets for all critical quality attributes. The responses selected for optimization were particle size (PS), polydispersity index (PDI), zeta potential (ZP), and encapsulation efficiency of SLB (EE). All responses were assigned a *target-is-best* goal, with the respective target values defined as follows: PS = 180 nm, PDI = 0.178, ZP = +35 mV, and EE = 90%. For EE, which did not yield a statistically significant regression model, the desirability function was constructed using experimental mean values rather than model-based predictions, ensuring that optimization was not biased by a nonpredictive model.

The optimization was conducted within the experimental design space defined by four independent variables. The optimal and global solution identified by the model corresponded to 0.0311 g/mL zein, 0.0060 g/mL chitosan, 30 min incubation and O:W ratio of 2.0, resulting in a composite desirability (D) of 0.8088 (Figure 7).

**Figure 7:**
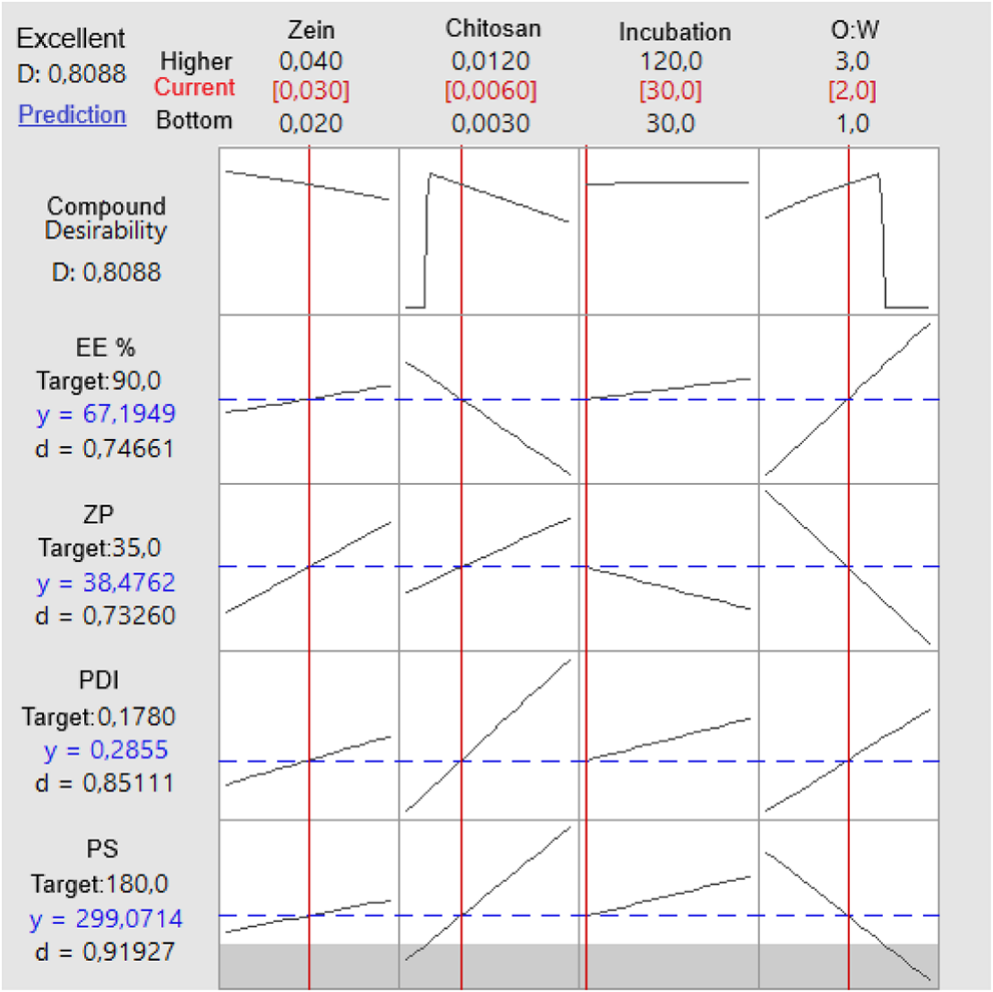
Desirability function plot for multiresponse, achieving a composite desirability of 0.8088.

Under these conditions, the predicted responses were PS = 299.07 nm (*d* = 0.919), PDI = 0.285 (*d* = 0.851), ZP = +38.4 mV (*d* = 0.732), and EE = 67.2% (*d* = 0.746). Although none of the responses fully met the specified targets, all exhibited individual desirability values above 0.50, indicating acceptable performance. The zeta potential was identified as the most limiting parameter, exceeding the target value by approximately 30%, while particle size and EE approached their respective goals with high desirability scores.

From a formulation standpoint, the optimized composition reflects a balance between polymer concentrations and O:W ratio, favoring colloidal stability and drug retention. The use of midrange chitosan content likely contributed to a positive surface charge well above the +35 mV target, enhancing electrostatic stabilization but deviating from the intended zeta-potential. The choice of an O:W ratio of 2.0 appears to be critical for maintaining low PDI values while sustaining acceptable EE. Incubation time showed minimal influence on the optimization outcome, allowing the selection of the shortest tested time (30 min) to improve process efficiency.

Overall, the desirability-based optimization yielded a robust balance between the evaluated responses, with a composite desirability close to 0.80. Future fine-tuning efforts may focus on adjusting chitosan concentration or modulating medium ionic strength to align zeta-potential with the target, while preserving the advantageous size distribution and encapsulation performance observed in the current optimized formulation.

The experimental validation of the desirability-based optimization revealed results that not only met but exceeded the predefined targets for the SLB-ZNP formulation intended for oral administration of the nanoparticle. The optimized nanoparticles presented a mean particle size of 144.6 nm (*d* = 1.11), which is substantially smaller than the 180 nm target. This nanoscale dimension is advantageous for oral delivery, as it increases the specific surface area available for dissolution, facilitates diffusion across the unstirred water layer of the gastrointestinal tract, and may enhance cellular uptake via endocytosis in enterocytes.^26,45^ Furthermore, a PDI of 0.187 (*d* = 0.01), indicates excellent colloidal uniformity, which is essential for reproducible dissolution profiles and predictable absorption kinetics.^46,47^

The zeta potential value of +40.3 mV (*d* = 4.0), which is higher than the +35 mV target, indicates strong electrostatic repulsion between particles, which minimizes aggregation during gastrointestinal transit. In the context of oral delivery, a pronounced positive surface charge, mainly conferred by the chitosan coating, can also promote mucoadhesion through electrostatic interaction with negatively charged mucins in the intestinal mucus layer. This interaction may increase the residence time of the nanoparticles in the absorptive regions of the small intestine, thereby enhancing the drug’s local concentration gradient and improving permeability.^48,49^

The EE of 90.7% (*d* = 2.61) greatly surpasses the 80% target, reflecting the strong affinity of SLB for the hydrophobic zein core and the stabilizing role of chitosan in preventing drug loss during the precipitation process. The high EE is particularly relevant for poorly soluble drugs like SLB, as it allows for higher payload delivery per dose, potentially reducing excipient burden and improving formulation cost-effectiveness.^50^

Overall, the experimental data confirm that the optimized formulation meets the physicochemical requirements for an effective oral nanocarrier system. The combination of sub-150 nm particle size, narrow distribution, high positive zeta-potential, and superior EE is expected to favor gastrointestinal stability, improve mucoadhesion, and enhance the oral bioavailability of SLB.^51,52^

The experimental results compared to the predicted model outputs suggest that the factorial design and desirability optimization captured the main parameter trends but underestimated synergistic effects between formulation variables. For instance, the combination of zein concentration at 0.0311 g/mL and chitosan at 0.0060 g/mL may have generated a more compact and stable nanoparticle interface than predicted, reducing particle size beyond model expectations. The higher zeta potential could result from more efficient chitosan adsorption at the particle surface during scaling from experimental design to validation, influenced by subtle variations in pH or ionic strength not accounted for in the model.

Likewise, the EE increase to 90.7% may reflect the optimized O:W ratio (2.0), which likely minimized drug loss to the aqueous phase during precipitation, in conjunction with enhanced hydrophobic interactions between zein and SLB. These synergistic effects, arising from polymer−drug affinity, interfacial stabilization, and process reproducibility, are not fully described by linear models, which explains the improved experimental performance. This outcome highlights the importance of experimental validation as a critical step following statistical optimization, particularly for complex colloidal systems such as protein-based nanoparticles.^53–55^

### 3.5 TEM Analysis

TEM was employed to investigate the morphology and structural organization of the optimized blank and SLB-ZNP. Figure 8a,b correspond to the blank nanoparticles, which exhibited predominantly spherical structures; however, a broader size distribution and the presence of irregularly shaped aggregates were evident. In higher-magnification images (Figure 8a), particles with heterogeneous diameters ranging approximately from 35 to 130 nm were observed, alongside amorphous clusters suggestive of partial particle coalescence or protein-rich domains. This morphological heterogeneity indicates a less compact interfacial organization in the absence of the bioactive compound, which may facilitate localized aggregation during particle formation or drying on the TEM grid.

**Figure 8:**
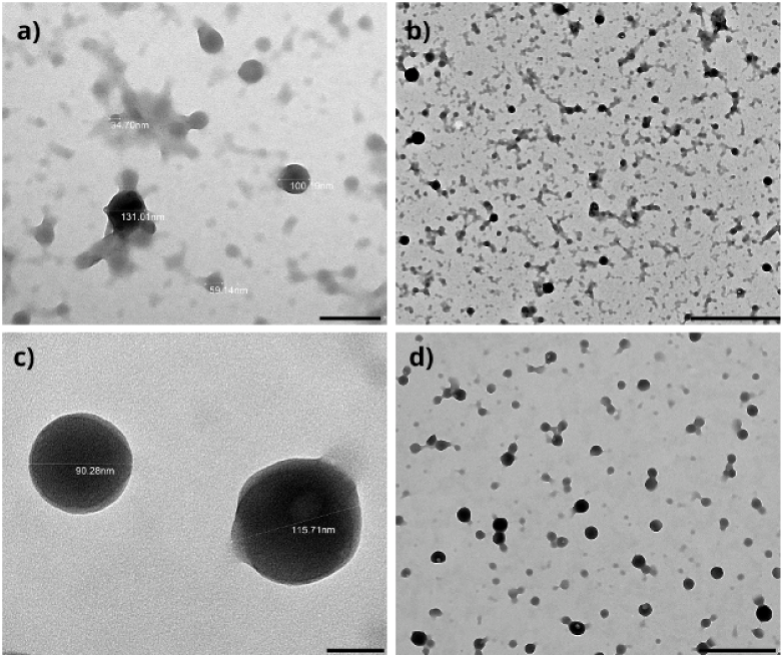
Transmission electron microscopy (TEM) micrographs of zein-based nanoparticles. Panels (a) and (b) show unloaded nanoparticles (ZNP) and panels (c) and (d) correspond to silibinin-loaded nanoparticles (SLB-ZNP).

In contrast, Figure 8c,d depict the SLB-ZNP, which showed a markedly improved morphological uniformity. These particles presented a well-defined spherical shape, smoother contours, and a narrower size range, with individual diameters predominantly between 90 and 120 nm. Importantly, no significant aggregation or irregular structures were detected in the loaded system, even at lower magnifications (Figure 8d), indicating enhanced colloidal stability and a more cohesive nanostructure upon drug incorporation.

The improved morphology of the loaded nanoparticles suggests that SLB plays an active role in stabilizing the zein−chitosan matrix, likely by promoting stronger hydrophobic interactions within the zein core and reinforcing electrostatic interactions at the particle interface. The presence of SLB may also contribute to a more homogeneous nucleation process during nanoprecipitation, resulting in more compact particles with reduced surface defects compared to the blank formulation.^9,27^

These TEM findings are consistent with the reduced particle size and increased surface charge observed experimentally and support the hypothesis that the optimized formulation conditions favor the formation of a dense and stable protein−polysaccharide shell. Moreover, the contrast between blank and loaded nanoparticles highlights the limitations of predictive statistical models, which may underestimate the structural reorganization induced by drug−matrix interactions that occur during experimental validation but are not explicitly captured within factorial design parameters.

### 3.6 Cytotoxicity over Tumor Cells

HeLa (cervical adenocarcinoma) and SiHa (cervical squamous carcinoma) cell lines were used as complementary in vitro models to evaluate the cytotoxic behavior of free SLB, blank ZNP, and SLB-ZNP (Figure 9). The tested concentration range (3161, 1581, and 790 μM, SLB-equivalent) was selected to enable discrimination of formulation-dependent effects under controlled in vitro conditions. Although higher than physiological exposure levels, such concentrations are commonly employed in exploratory cytotoxicity screening to identify relative differences between formulations. All experiments were performed with limited biological replication (n = 2), and results are therefore interpreted qualitatively.

**Figure 9:**
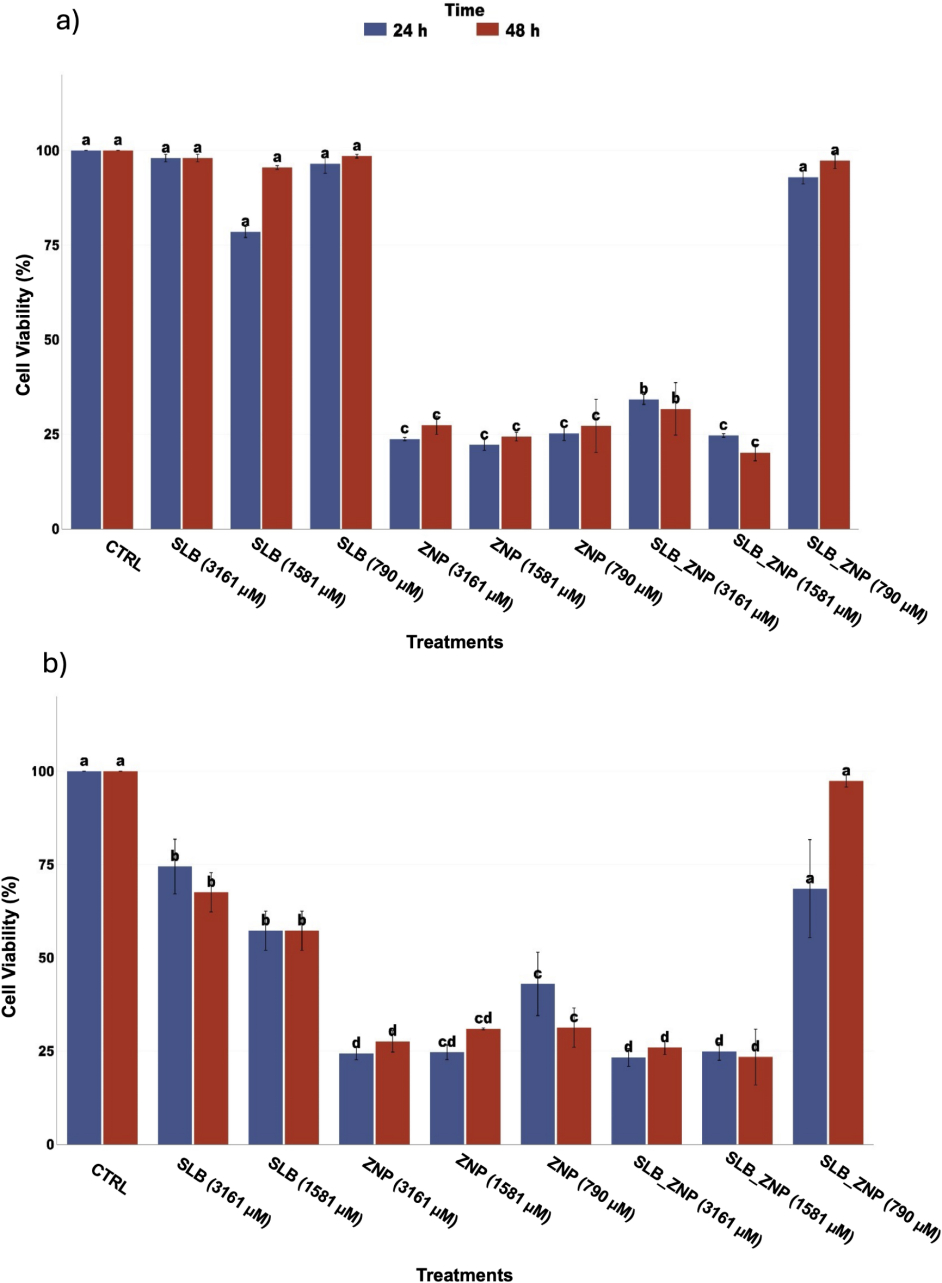
Cell viability of cervical cancer cell lines after 24 and 48 h of exposure to free silibinin (SLB), blank nanoparticles (ZNP), and silibinin-loaded nanoparticles (SLB-ZNP) at SLB-equivalent concentrations of 3161, 1581, and 790 μM: (a) HeLa cells and (b) SiHa cells.

Blank ZNP induced a marked reduction in cell viability in both cell lines across all tested concentrations and exposure times. The effect was largely concentration-independent and exhibited limited time dependence, suggesting rapid onset of cytotoxicity. This behavior is consistent with the high positive surface charge of the zein−chitosan carrier, which may promote strong electrostatic interactions with negatively charged cellular membranes. Such interactions can lead to membrane destabilization and cellular stress, as widely described for cationic nanostructures.^56,57^ Notably, HeLa cells appeared more sensitive to ZNP exposure than SiHa cells, indicating cell line−dependent tolerance to the cationic carrier.

Encapsulation of SLB significantly altered the cytotoxic profile of the nanoparticle system. In HeLa cells, SLB-ZNP consistently exhibited lower cytotoxicity than blank ZNP at equivalent SLB-equivalent concentrations, indicating that drug incorporation mitigates nonspecific carrier-associated toxicity. Interestingly, the strongest reduction in HeLa cell viability for SLB-ZNP was observed at 1581 μM, whereas the highest concentration (3161 μM) did not produce a proportionally greater effect, revealing a nonmonotonic concentration−response pattern.

In SiHa cells, the mitigating effect of SLB incorporation was particularly evident at 790 μM. At this concentration, blank ZNP reduced cell viability to approximately 50% (24 h) and 40% (48 h), whereas SLB-ZNP maintained substantially higher viability levels (∼75% at 24 h and ∼100% at 48 h). This clear difference demonstrates that SLB loading attenuates the nonspecific cytotoxicity associated with the zein−chitosan carrier rather than producing comparable viability profiles. At higher concentrations (1581 and 3161 μM), SiHa cells exhibited a clearer concentration-dependent trend for SLB-ZNP, with progressively stronger antiproliferative effects at increasing SLB-equivalent concentrations.

Free SLB exhibited modest and cell line−dependent antiproliferative activity. HeLa cells showed limited sensitivity to free SLB across the tested concentrations, whereas SiHa cells demonstrated a more pronounced response at higher concentrations. These findings indicate that intrinsic cellular characteristics contribute substantially to the observed biological response.^58,59^

Taken together, the data indicate that cytotoxic behavior in this system is primarily formulation-driven. Blank zein−chitosan nanoparticles exhibit nonspecific cytotoxicity likely associated with their cationic surface properties. SLB incorporation mitigates this carrier-associated toxicity, particularly in HeLa cells and at lower concentrations in SiHa cells. Additionally, the magnitude and pattern of response are strongly dependent on the cellular model. These findings highlight that the biological effects observed are governed by nanoparticle physicochemical properties and drug−matrix interactions rather than solely by drug-mediated cytotoxic mechanisms.

## 4 Conclusion

Collectively, the results of this study demonstrate the successful formulation and systematic optimization of zein−chitosan nanoparticles encapsulating SLB, yielding a nanocarrier system with well-defined physicochemical properties, colloidal stability, and consistent in vitro behavior. The application of a full 2^4^ factorial design enabled a quantitative assessment of formulation and process variables and their interactions, providing a rational framework for nanoparticle engineering. The optimized nanoparticles exhibited favorable size distribution, high positive surface charge, and elevated encapsulation efficiency. Cytotoxicity studies revealed that blank zein−chitosan nanoparticles induce nonspecific cellular effects likely associated with their cationic surface properties, while SLB incorporation attenuates this carrier-associated toxicity and modulates the biological response in a cell line−dependent manner. These findings highlight the importance of considering formulation-driven effects when interpreting in vitro cytotoxicity of protein−polysaccharide nanocarriers. No pharmacokinetic or in vivo evaluations were performed in this study; therefore, potential improvements in oral bioavailability and translational applicability should be regarded as hypotheses requiring further investigation. Future studies will focus on in vivo validation and additional mechanistic analyses to determine whether the optimized formulation translates into enhanced oral absorption and therapeutic performance.

## Supplementary Material



## Notes

Statistical validation data for the factorial design models, including histograms and normal probability plots of residuals for particle size (Figure S1), polydispersity index (Figure S2), and zeta potential (Figure S3), confirming the adequacy of the regression assumptions (PDF)
