# Supplementary material for: Factorial Design−Driven Optimization of Zein−Chitosan Nanoparticles for Oral Delivery of Silibinin
Source: ACS Omega. 2026 Mar 6;11(10):16673–86. doi: 10.1021/acsomega.5c13167 (PMC13000786; doi:10.1021/acsomega.5c13167)
Supplement: Supplementary file 1 [file ao5c13167_si_001.pdf]

Factorial Design–Driven Optimization of Zein–Chitosan Nanoparticles for Oral Delivery of Silibinin

Rafaelle de Sertorio dos Santos[a]; Ariane Krause Padilha Lorenzett[a]; Gabriela Casa Grande de Matos[b]; Patrícia de Souza Bonfim-Mendonça[b]; Vanderlei Aparecido de Lima[c]; Rubiana Mara Mainardes[a*]

[a]Laboratory of Nanostructured Formulations, Midwest State University – UNICENTRO, Alameda Élio Antonio Dalla Vecchia St., 838, Guarapuava 85040-167, PR, Brazil

[b] Pharmacy Department, Maringa State University - UEM, Avenida Colombo, St.,5790, Jardim Universitário, Maringá, 87020-900, PR, Brazil

[c]Chemistry Department, Federal Technological University of Paraná - UTFPR, Via do Conhecimento, s/n - KM 01 - Fraron, Pato Branco - PR, 85503-390, Pato Branco PR, Brazil

*mainardes@unicentro.br

Supplemental Material

S1. (a) Histogram of particle size residuals (b) Particle size normal probability residual plot.

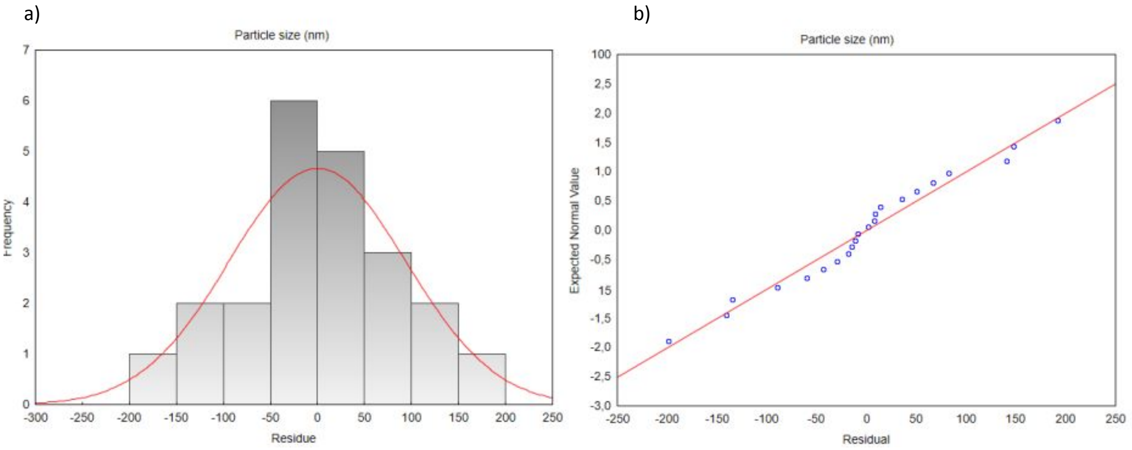

S2. Histogram of Polydispersity Index residuals (b) Polydispersity Index normal probability residual plot.

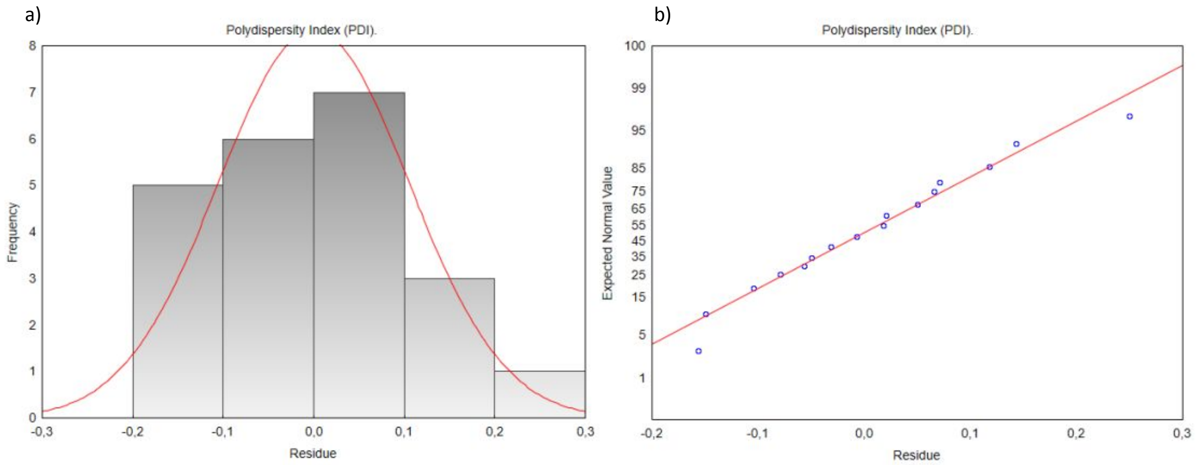

S3. Histogram of Zeta Potential residuals (b) Zeta Potential normal probability residual plot.

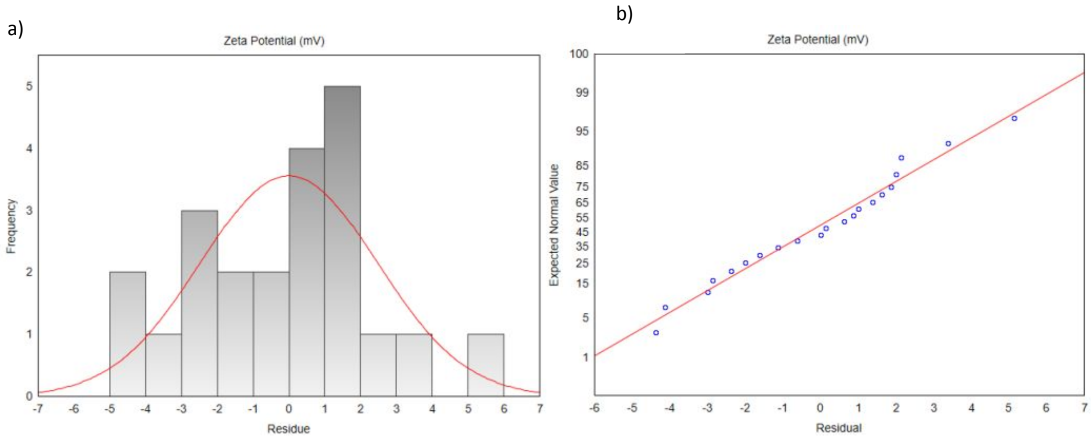
a)
Zeta Potential (mV)
Frequency
Residue
b)
Zeta Potential (mV)
Expected Normal Value
Residual
